# Clinical evidence for low-molecular-weight collagen peptides in tendon and ligament tears: a systematic review with narrative synthesis

**DOI:** 10.3389/fmed.2026.1891011

**Published:** 2026-08-14

**Authors:** Luca Latini, Francesco Porta, Nicolò Vitale, Florentin Ananu Vreju, Emilio Filippucci

**Affiliations:** 1 Centro Medico e Fisioterapia “Salute e Benessere”, Senigallia, Ancona, Italy; 2 Interdisciplinary Pain Medicine Unit, Santa Maria Maddalena Hospital, Occhiobello, Italy; 3 Centro Medicina Riabilitativa “CMR”, Adrano, Catania, Italy; 4 Department of Rheumatology, University of Medicine and Pharmacy of Craiova, Craiova, Romania; 5 Rheumatology Unit, Department of Clinical and Molecular Sciences, Polytechnic University of Marche, Jesi, Ancona, Italy

**Keywords:** ACL reconstruction, collagen peptides, hydrolyzed collagen, ligament tear, low-molecular-weight peptides, rotator cuff tear, tendon tear, ultrasound-guided injection

## Abstract

**Background:**

Low-molecular-weight hydrolyzed collagen peptides are increasingly used as adjuncts in musculoskeletal medicine, but their clinical role in tendon and ligament injuries remains unclear.

**Objective:**

To evaluate clinical evidence on oral or injectable hydrolyzed collagen peptides, or related low-molecular-weight peptide preparations, in tendon and ligament injuries and post-repair/reconstruction settings.

**Methods:**

A systematic review with narrative synthesis was conducted using PubMed and a mirrored Scopus strategy through 17 March 2026. Eligible studies were human clinical studies assessing hydrolyzed collagen peptides or clearly related low-molecular-weight peptide formulations in tendon tears, ligament tears, or postoperative repair/reconstruction. Closely related disorders were included only as contextual evidence when direct injury-specific data were limited.

**Results:**

Six studies met the eligibility criteria. Three provided direct evidence in tear or post-surgical populations: one pilot study of injectable low-molecular-weight peptides in partial supraspinatus tendon tears, one randomized trial after ACL reconstruction, and one randomized trial after rotator cuff repair. Three additional studies provided indirect evidence in Achilles tendinopathy, chronic ankle instability, and rotator cuff tendinopathy. Overall, collagen-based interventions were associated with improvements in pain, function, analgesic use, or imaging-related outcomes. However, most oral interventions were multicomponent, and only one direct tear study evaluated a clearly defined low-molecular-weight peptide formulation.

**Conclusions:**

Hydrolyzed collagen-derived peptides show a cautiously positive signal as adjuncts to rehabilitation or postoperative care. However, evidence in tendon and ligament tears remains sparse, heterogeneous, and low certainty. Further blinded, placebo-controlled trials using well-characterized formulations and clinically meaningful outcomes are needed.

## 1 Introduction

Tendons and ligaments are collagen-rich connective tissues whose healing is often slow, mechanically incomplete, and clinically variable. This has stimulated interest in nutritional and injectable adjuncts that may support extracellular matrix repair and improve symptoms during rehabilitation in post-operative recovery.

Hydrolyzed collagen is biologically attractive because oral ingestion yields measurable hydroxyproline-containing peptides such as Gly-Pro-Hyp and Pro-Hyp in the circulation, providing a plausible mechanistic rationale for downstream tissue effects (1).

Previous studies showed that specific collagen-derived peptides like Pro-Hyp, and Hyp=Gly can be detected in the bloodstream of patients after ingestion, suggesting their systemic bioavailability and biological activity (2).

Experimental data also suggest that these peptides may influence fibroblast activity and extracellular matrix turnover and remodeling (3).

However, biological plausibility does not establish clinical efficacy in the healing of actual tendon or ligament tears.

The clinical literature remains small and heterogeneous. Reviews of nutritional supplementation in tendinopathy and broader meta-analyses of collagen peptide supplementation suggest possible benefits for pain, function, and tendon morphology, but they also emphasize low certainty and a lack of injury-specific trials (4–6).

The aim of the present review was therefore to assess the impact of low-molecular-weight peptides from hydrolyzed collagen in patients with tendon and ligament tears. Because direct tear-specific studies remain few, adjacent postoperative and closely related clinical evidence was also considered, while keeping a clear distinction between direct and indirect evidence.

## 2 Methods

**Figure 1:**
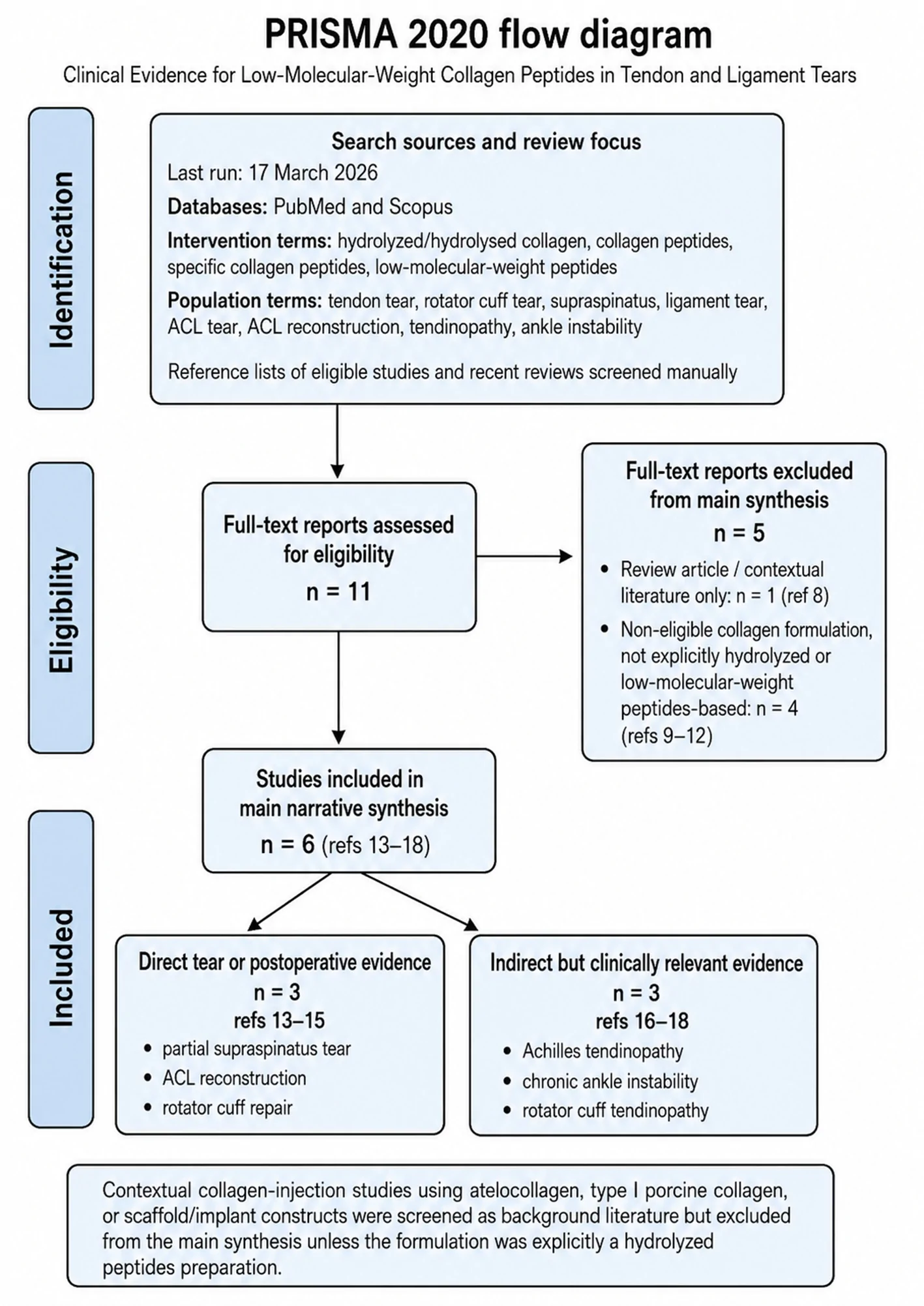
PRISMA 2020 flow diagram.

This manuscript was structured as a systematic review with narrative synthesis. Core PRISMA 2020 reporting principles were used to organize the search, eligibility criteria, extraction, and reporting framework (7) (Figure 1). A meta-analysis was not attempted because of marked clinical and methodological heterogeneity across formulations, routes of administration, co-interventions, outcome measures, and follow-up durations.

### 2.1 Information sources and search strategy

The literature search was updated through 17 March 2026 in PubMed and by using a concept-matched Scopus strategy. The intervention block included “hydrolyzed collagen”, “hydrolysed collagen”, “collagen peptides”, “specific collagen peptides”, and “low-molecular-weight peptides”. The population block included “tendon tear”, “rotator cuff tear”, “supraspinatus tear”, “ligament tear”, “ACL tear”, “ACL reconstruction”, “tendinopathy”, and “ankle instability”. Reference lists of eligible studies and recent reviews were also screened manually.

The review focused on hydrolyzed collagen-derived peptides and clearly described low-molecular-weight peptides preparations. During screening, a broader shoulder literature on collagen injections was also identified, including atelocollagen and type I porcine collagen studies; these were retained only as contextual background when the formulation was not explicitly hydrolyzed or peptides-based (8–12).

### 2.2 Eligibility criteria

Studies were eligible for the main synthesis when they: (1) included human participants with tendon or ligament tears, or patients undergoing reconstructive surgery for tendon or ligament injuries; (2) evaluated oral hydrolyzed collagen peptides or injectable low-molecular-weight collagen-derived peptides formulations; and (3) reported clinical, functional, imaging, or safety outcomes.

Studies in related disorders such as chronic tendinopathy or chronic ankle instability were retained for contextual synthesis only when the intervention was clinically comparable and direct tear-specific evidence was too limited to stand alone. Preclinical studies, protocols without results, narrative reviews, scaffold or implant studies, and studies using collagen exclusively as native/non-hydrolyzed biomaterial rather than a peptides intervention were excluded from the main synthesis.

### 2.3 Data extraction and qualitative appraisal

For each eligible study, the following data were extracted: design, population, intervention, comparator, follow-up, main outcomes, and the main threats to validity. Because pooled analysis was not appropriate, risk of bias was assessed qualitatively with emphasis on randomization, blinding, placebo/control use, intervention specificity, completeness of reporting, and objectivity of the primary endpoints.

## 3 Results

The updated search confirmed that the literature specific to hydrolyzed collagen-derived peptides in tendon and ligament tears remains very small. Six studies met the main eligibility criteria for the narrative synthesis (13–18) and their main characteristics are reported in Table 1. Additional collagen-based shoulder studies were identified during screening, including atelocollagen, type I porcine collagen, and combination-injection studies in partial-thickness rotator cuff tears or supraspinatus tendinopathy, but these were not included in the main synthesis when the formulation was not explicitly hydrolyzed or low-molecular-weight peptides-based (8–12).

**Table 1:** Characteristics of studies included in the main narrative synthesis.

Reference	Study design	Population	Intervention/comparator	Main findings	Main limitation/directness
Latini et al. (13)	Pilot single-arm study	Twenty-one patients with symptomatic partial supraspinatus tendon tears	Two ultrasound-guided intratendinous injections of 5 mg/1 ml low-molecular-weight peptides from hydrolyzed bovine collagen; no comparator	VAS-P and SPADI improved over 12 weeks; one tear ultrasound progression reported	Uncontrolled exploratory design; direct tear ultrasound evidence
Lopez-Vidriero et al. (14)	Randomized controlled trial	Seventy two patients after ACL reconstruction	Oral progen supplement (hydrolyzed collagen peptides + plasma proteins + HA/chondroitin + vitamin C) vs. rehabilitation alone	Lower analgesic use, fewer physiotherapy sessions, better IKDC at 60 days, and more advanced MRI graft maturation at 90 days	Open-label multicomponent formulation; direct post-tear surgical evidence
Gumina et al. (15)	Prospective randomized study	Ninety patients after arthroscopic repair of large posterosuperior rotator cuff tears	Oral supplement containing hydrolyzed type I collagen + arginine alpha-ketoglutarate + MSM + bromelain vs. no supplement	Lower postoperative pain and slightly better repair integrity	Multicomponent formulation and limited attribution to collagen; direct post-tear surgical evidence
Praet et al. (16)	Randomized double-blind crossover pilot	Twenty patients with chronic mid-portion Achilles tendinopathy	Specific collagen peptides plus calf-strengthening program vs. placebo crossover	Earlier VISA-A improvement with collagen peptides; no adverse events reported	Small pilot in non-tear population; indirect evidence
Dressler et al. (17)	Randomized double-blind placebo-controlled trial	Fifty athletes with chronic ankle instability	5 g/day specific collagen peptides vs. placebo for 6 months	Better CAIT and FAAM-G scores and fewer reinjuries at follow-up; no clear arthrometer difference	Related ligament dysfunction rather than acute tear healing; indirect evidence
Buda et al. (18)	Multicentric prospective observational study	Seventy one patients with rotator cuff tendinopathy	Two subacromial injections of hydrolyzed collagen (< 3 kDa); no comparator	Marked pain reduction and improved Constant Score and SST through 6 months; no major adverse events	Uncontrolled heterogeneous shoulder population; indirect evidence

ACL, anterior cruciate ligament; CAIT, cumberland ankle instability tool; HA, hyaluronate acid; IKDC, International Knee Documentation Committee; FAAM-G, foot and ankle ability measure-German; MSM, methylsulfonylmethane; SPADI, shoulder pain and disability index; SST, simple shoulder test; VAS-P, visual analogue score for pain; VISA-A, Victorian Institute of Sport Assessment–Achilles.

### 3.1 Evidence in tear and post-surgical populations

Only one study directly evaluated a declared low-molecular-weight peptides preparation in a tendon tear population. Latini et al. (13) treated 21 patients with symptomatic partial supraspinatus tendon tears using two ultrasound-guided intratendinous injections of a 5 mg/1 ml low-molecular-weight peptides solution derived from hydrolyzed bovine collagen. Pain and SPADI scores improved at 12 weeks, but the study was uncontrolled and exploratory.

Two additional studies addressed postoperative populations in which collagen-containing supplements were administered during tendon or ligament healing. In ACL reconstruction, López-Vidriero et al. (14) reported lower analgesic consumption, fewer physiotherapy sessions, better short-term IKDC scores, and more advanced MRI graft maturation in the supplemented arm; however, the product also contained plasma proteins, hyaluronic acid/chondroitin sulfate, and vitamin C. In patients undergoing arthroscopic repair of large posterosuperior rotator cuff tears, Gumina et al. (15) observed lower postoperative pain and somewhat better repair integrity with a supplement containing hydrolyzed type I collagen plus arginine alpha-ketoglutarate, methylsulfonylmethane, and bromelain.

### 3.2 Clinically relevant contextual evidence

Three studies were retained as contextual evidence because direct tear-specific trials were scarce. In chronic mid-portion Achilles tendinopathy, Praet et al. (16) found that specific collagen peptides combined with calf-strengthening exercises accelerated clinical improvement compared with placebo crossover, although the study was small and not focused on a tear population.

In athletes with chronic ankle instability, Dressler et al. (17) reported better subjective ankle stability and fewer reinjuries after 6 months of oral supplementation with 5 g/day specific collagen peptides, but objective arthrometer-derived ankle stiffness was unchanged. In rotator cuff tendinopathy, Buda et al. (18) described clinically relevant improvements after two subacromial injections of hydrolyzed collagen with molecular size < 3 kDa; the study was prospective but uncontrolled and included a mixed tendinopathy population rather than a defined tear cohort.

### 3.3 Qualitative risk of bias

Overall certainty of evidence was low (Table 2). The main sources of bias were small sample size, lack of placebo control, absence of blinding, inaccurate assessment of the tendon and ligament tear, multicomponent formulations, and reliance on short-term patient-reported outcomes. Even when randomization was present, attribution of efficacy to hydrolyzed collagen peptides alone was usually impossible because collagen was co-administered with other active ingredients (14, 15).

**Table 2:** Qualitative appraisal of internal validity.

Study	Tool and assessed result	Domain/item-level judgment and justification	Overall judgment
Latini et al. (13)	Revised JBI quasi-experimental tool; VAS pain/SPADI at 12 weeks	Q1 Y; Q2 N; Q3 NA; Q4 NA; Q5 N; Q6 NA; Q7 Y; Q8 U; Q9 U. Uncontrolled pre-post design; only one pre-intervention assessment; one participant did not complete T2; the paired/repeated-measures approach was not explicitly specified	High risk of bias^*^
López-Vidriero et al. (14)	RoB 2, parallel-group; VAS/IKDC at 90 days	D1 low; D2 some concerns; D3 low; D4 high; D5 some concerns. Centralized randomization and balanced attrition, but the trial was open-label without placebo; participants and treating staff knew allocation, and the principal outcomes were patient reported	High
Gumina et al. (15)	RoB 2, parallel-group; postoperative pain at 6 months	D1 some concerns; D2 some concerns; D3 some concerns; D4 high; D5 some concerns. The report states randomization but gives insufficient detail on sequence generation/allocation concealment; no placebo or blinding was reported for the subjective pain outcome, and attrition/reporting information was limited	High
Praet et al. (16)	RoB 2, crossover; VISA-A at 6 months	D1 low; DS some concerns; D2 low; D3 some concerns; D4 low; D5 low. computerized block randomization, allocation concealment, and double blinding were reported. A true washout was not feasible, although carryover was modeled; 18/20 participants provided 6-month data	Some concerns
Dressler et al. (17)	RoB 2, parallel-group; CAIT/FAAM-G at 6 months	D1 high; D2 high; D3 high; D4 low; D5 some concerns. Important baseline imbalance was present in the principal subjective outcomes; 10/60 randomized participants were excluded after randomization, including for non-adherence, and the analysis was based on completers	High
Buda et al. (18)	Revised JBI quasi-experimental tool; VAS/constant score/SST at 6 months	Q1 Y; Q2 N; Q3 NA; Q4 NA; Q5 N; Q6 NA; Q7 Y; Q8 U; Q9 U. Uncontrolled multicenter pre-post study; only one true pre-intervention assessment; participant retention and the handling of repeated measurements were not reported in sufficient detail	High risk of bias^*^

Risk-of-bias assessment using validated design-specific tools.

RoB 2 domains: D1, randomization process; D2, deviations from intended interventions; D3, missing outcome data; D4, measurement of the outcome; D5, selection of the reported result; DS, bias arising from period and carryover effects in the crossover trial. JBI items: Q1, temporal precedence; Q2, control group; Q3, similarity of compared participants; Q4, comparable care; Q5, multiple pre/post measurements; Q6, identical outcome measurement across comparisons; Q7, reliable outcome measurement; Q8, complete follow-up; Q9, appropriate statistical analysis. Y, yes; N, no; U, unclear; NA, not applicable. ^*^The revised JBI tool does not prescribe an algorithmic overall category; the overall judgment shown here is a review-level judgment based on the pattern and severity of item-level limitations.

## 4 Discussion

The main message of this review is that clinical enthusiasm currently exceeds evidential strength. Only one study specifically evaluated a declared low-molecular-weight hydrolyzed collagen peptides preparation, and it was a small uncontrolled pilot (13). The two other direct studies were postoperative trials using multicomponent oral supplements, making it impossible to isolate the specific contribution of hydrolyzed collagen peptides (14, 15).

Even so, the direction of effect across studies is reasonably consistent. Whether delivered orally or by injection, collagen-based interventions tended to improve pain, patient-reported function, analgesic use, or imaging-related findings. This pattern is compatible with the broader literature suggesting that collagen-derived peptides may be most useful when paired with rehabilitation, mechanical loading, or postoperative tissue remodeling rather than used as stand-alone therapy (4, 5).

A second important issue is the inconsistent characterization of peptides size. Low-molecular-weight status was clearly declared in the injectable shoulder studies retained in the main synthesis (13, 18). In contrast, most oral studies referred more generically to “hydrolyzed collagen peptides” or “specific collagen peptides” without detailed molecular-weight distribution data (14–17). For a review centered on low-molecular-weight peptides, this is a major limitation in the literature.

The updated search also underscored how much adjacent evidence falls just outside the strict formulation question. A growing shoulder literature describes atelocollagen or type I collagen injections for partial-thickness rotator cuff tears and supraspinatus tendinopathy, including prospective comparative studies and randomized trials (8–12). These studies support a broader signal that collagen-based injectables may improve pain and, in some cases, imaging outcomes. However, because these formulations are not clearly hydrolyzed peptides preparations, they should not be conflated with evidence for low-molecular-weight hydrolyzed collagen peptides.

Clinically, the current evidence supports hydrolyzed collagen-derived peptides only as possible adjuncts to established rehabilitation or postoperative care. There is still no robust proof that these products reduce rerupture rates, accelerate return to sport with certainty, or reliably improve objective biomechanical healing endpoints in acute tendon or ligament tears. At present, the most defensible clinical position is one of cautious adjunctive use rather than evidence-based standard care.

The concept of synergistic effect between collagen supplementation and mechanical loading in stimulating collagen synthesis is supported by experimental evidence; mechanistic data in healthy adults further support reinforcing the idea that these interventions are unlikely to be efficient as stand-alone therapies (19).

## 5 Limitations

This review has several limitations. First, the evidence base itself is very small and heterogeneous. Second, most oral interventions were multicomponent formulations, which prevents confident attribution of benefit to collagen peptides alone. Third, outcomes were commonly short-term and patient-reported, with limited information on structural healing durability, reinjury, or return to sport. Fourth, the review used a focused two-database update with manual citation tracking and qualitative appraisal rather than a full meta-analytic workflow. Finally, the formulation-focused eligibility criteria intentionally excluded part of the broader collagen-injection literature, which improves conceptual specificity but reduces breadth.

## 6 Conclusions

Hydrolyzed collagen-derived peptides, including low-molecular-weight formulations, have a plausible biological rationale and a cautiously favorable signal in tendon and ligament clinical care. Nevertheless, evidence specific to tendon and ligament tears remains limited to small pilots, heterogeneous postoperative studies, and indirect supportive trials in related disorders.

Furthermore, the low-molecular-weight framing of this review is firmly supported by explicit molecular-weight characterization in only two of the six included studies (13, 18), whereas the remaining studies used broader descriptions such as hydrolyzed collagen peptides or specific collagen peptides.

Future research should prioritize blinded, placebo-controlled trials with clearly characterized low-molecular-weight formulations and clinically meaningful endpoints such as structural healing, incidence of rerupture, return to daily work and sport activities, imaging guidance in performing injections and durable patient-important outcomes. Until then, these products should be regarded as promising adjunctive therapies rather than established standards of care.

## Appendix 1. Search strategies

**PubMed search (last run 17 March 2026):** (“hydrolyzed collagen”[Title/Abstract] OR “hydrolysed collagen”[Title/Abstract] OR “collagen peptides”[Title/Abstract] OR “specific collagen peptides”[Title/Abstract] OR “low-molecular-weight peptide*”[Title/Abstract]) AND (“tendon tear”[Title/Abstract] OR “rotator cuff tear”[Title/Abstract] OR supraspinatus[Title/Abstract] OR “ligament tear”[Title/Abstract] OR “ACL tear”[Title/Abstract] OR “ACL reconstruction”[Title/Abstract] OR tendinopathy[Title/Abstract] OR “ankle instability”[Title/Abstract]) **Scopus strategy (concept-matched):** TITLE-ABS-KEY (“hydrolyzed collagen” OR “hydrolysed collagen” OR “collagen peptides” OR “specific collagen peptides” OR “low-molecular-weight peptides”) AND TITLE-ABS-KEY (“tendon tear” OR “rotator cuff tear” OR supraspinatus OR “ligament tear” OR “ACL tear” OR “ACL reconstruction” OR tendinopathy OR “ankle instability”)

Of note, broader collagen-injection studies using atelocollagen, type I porcine collagen, or scaffold/implant constructs were screened as contextual literature but were excluded from the main synthesis unless the formulation was explicitly a hydrolyzed peptides preparation.
